# Editorial: X-linked retinoschisis: mechanisms and therapies

**DOI:** 10.3389/fmed.2024.1523215

**Published:** 2024-12-18

**Authors:** Alina V. Dumitrescu, Mary Elizabeth Hartnett, Arlene V. Drack

**Affiliations:** ^1^Department of Ophthalmology and Visual Sciences, The University of Iowa, Iowa City, IA, United States; ^2^Department of Ophthalmology, Stanford University, Stanford, CA, United States; ^3^Institute for Vision Research, Department of Ophthalmology and Visual Sciences, The University of Iowa, Iowa City, IA, United States

**Keywords:** X-linked retinoschisis, gene therapy, retina, carbonic anhydrase inhibitors, OCT

X-linked retinoschisis (XLRS) is the most common hereditary juvenile macular degeneration in males, resulting in significant vision impairment. Generally, disease progression is slow but can lead to consequential visual loss over time. Pathogenic variants in one gene (*RS1*) cause XLRS, making it an important clinical target for developing treatments.

This Research Topic features 7 original articles that address the natural history, mechanism, and possible treatment of XLRS.

## Clinical features and disease progression

There is significant variability in the severity of symptoms, with some patients with XLRS experiencing relatively mild vision loss while others suffer from more severe forms. The reasons for variability, even among patients with the same variant, remain unknown, complicating prognosis especially for young patients. Two studies in this Research Topic explore these challenges. Both sought to elucidate functional and structural outcomes by analyzing clinical records of patients with molecularly confirmed XLRS.

Fenner et al. examined 52 patients across 33 families, finding that XLRS commonly presents around age 11 years, although symptoms started around age 5. Almost all patients had macular schisis, while inferotemporal peripheral retinoschisis was noted in 40.4%. When analyzing optic coherence tomography (OCT) images, many patients showed outer retinal loss by age 20, with outer retinal atrophy (ORA) and decreased visual acuity (VA) by age 40. Retinal detachment, occurring in 7.7% of this cohort, was linked to poorer visual outcomes, highlighting the need for effective prevention and management strategies. *RS1* null genotypes and central subfield thickness (CST) ≥ 450 microns at presentation increased the risk of at least moderate visual impairment at final follow-up independent of age at onset, initial ORA, or previous retinal detachment (Fenner et al.).

Kiraly et al. analyzed 118 eyes of 59 XLRS patients (average age 24), finding a complex relationship between retinal morphology and visual function. While CST is used in monitoring patients with XLRS, it doesn't always correlate with visual acuity. The authors found a strong symmetry of CST and macular volume between eyes within individual patients, suggesting OCT CST may be a more reliable endpoint than VA for comparison between a patient's two eyes in clinical trials (Kiraly et al.).

## Gene therapy and other treatments

Intravitreal gene replacement therapy has shown promise for improving retinal structure and function in preclinical models and early clinical trials. However, inflammation following the procedure has limited VA gains (1, 2). A challenge is the variability in patient responses influenced by factors such as *RS1* mutation type and disease stage. Individual immune responses seem to play a role in the disease pathogenicity and may impact treatment outcomes.

In this Research Topic, Hassan et al. treated *Rs1*-KO mice with subretinal injections of different doses of rAAV2tYF-CB-h*RS1* vector previously used in human clinical trials as an intravitreal injection. Murine subretinal injections yielded dose-dependent reconstitution of human RS1 protein and markedly improved retinal function, especially in light-adapted electroretinography (ERG). OCT showed reduced cystic cavity size, and immunohistochemistry demonstrated considerable cone rescue. The subretinal injection was well tolerated, even in eyes with significant retinal layer schisis. The visually guided swim assay indicated improved functional vision as well, suggesting that preclinical findings may translate to human trials (Hassan et al.).

Unexpectedly, Gehrke, Thompson et al. reported that subretinal injections of a hypertonic buffer also reduced cyst severity and increased cone-dominant ERG amplitudes. In the long term, treated eyes showed significantly higher cone counts than untreated eyes, suggesting that early cyst resolution may enhance cone signaling through an osmolarity-dependent mechanism and improve cone viability, providing valuable insight for future XLRS therapies (Gehrke, Thompson et al.).

Additionally, while the gene *Casp1*, has been reported to be upregulated in *Rs1*-KO mice (3), Gehrke, Pandey et al. demonstrated that *Rs1-Casp1* double-deficient mice exhibited no improvement in photoreceptor function or retinal structure, indicating that *Casp1* may not play a primary role in disease progression but is instead secondarily upregulated.

These findings highlight the potential benefits of subretinal over intravitreal gene therapy for XLRS and suggest possible alternative treatments involving osmolarity.

Topical or systemic carbonic anhydrase inhibitors (CAIs) have reportedly shown efficacy in reducing cysts and modestly enhancing vision in XLRS patients (4–6). Wey et al., report in this Research Topic that CAI use reduced median CST without significantly improving VA in 26 eyes. No difference was found between topical and systemic delivery. Nineteen eyes had peripheral schisis; two of these resolved on CAIs. 10 patients presented with retinal detachment with a median VA 20/150 and a final VA 20/400. Clinical stability over 2.9 year median follow-up was noted (Wey et al.). Large, prospective trials are necessary to assess CAIs' potential to prevent complications and preserve cone survival long term.

## Grading peripheral retinoschisis

Retinal detachment in XLRS is a serious complication that may affect visual outcomes. Despite its importance, the mechanisms underlying retinal detachment in XLRS are poorly understood, thwarting efforts to prevent it. An intriguing finding reported in this Research Topic by Nakajima et al. is that occult retinoschisis, in which retinal splitting is observed in the ganglion cell layer (GCL) on OCT but is not detectable on ophthalmoscopic examination, may occur in the retinal periphery. The authors propose a Grading Protocol for peripheral schisis, and hypothesize that occult peripheral schisis in the GCL may precede or precipitate retinal detachment. This hypothesis is worthy of further study, making widefield OCT monitoring essential for detection (Nakajima et al.).

## Conclusion

The original articles in this Research Topic highlight key findings and actionable insights on XLRS progression. VA typically remains stable in XLRS patients until their 20s, after which ORA leads to VA decline. CST is initially thicker than normal due to cysts, then thins below normal with age and ORA. Preventing this inevitable progression and reducing the risk of retinal detachments are the primary challenges for future research.

ORA correlates more strongly with reduced VA than CST, though CST is more symmetric between eyes. This suggests CST may serve as an inter-eye endpoint in trials where only one eye is treated, with ORA and VA considered for longer-term assessments. Null mutations and initial CST ≥450 microns portend worse VA outcomes, offering potential criteria for randomization in clinical trials. Peripheral and GCL schisis should be explored in larger studies.

Two studies using XLRS mouse models show that reducing cyst size can improve cone viability and function over time, raising the question of whether CAIs or other treatments that reduce cysts could also decrease long-term atrophy in humans.

Subretinal gene replacement or hypertonic saline injections improved short and long-term cone function in mice. Current human subretinal gene therapy trials may reveal the best treatment approach. Over time, XLRS leads to retinal thinning and reduced rod/cone ERG amplitudes, suggesting that XLRS evolves into a retinal degeneration and underscoring the importance of careful patient selection for trials as well as clinical counseling.

While the challenges are many, the concentrated efforts of physicians and researchers hold promise for future XLRS breakthroughs.
